# Physiological and pharmacological aspects of the vas deferens—an update

**DOI:** 10.3389/fphar.2013.00101

**Published:** 2013-08-22

**Authors:** David S. Koslov, Karl-Erik Andersson

**Affiliations:** ^1^Wake Forest Baptist Medical Center, Medical Center BoulevardWinston-Salem, NC, USA; ^2^Institute for Regenerative Medicine, Wake Forest University School of MedicineWinston-Salem, NC, USA

**Keywords:** vas deferens, smooth muscle, adrenergic receptors, contraction, fertility, purinergic receptors

## Abstract

The vas deferens, a muscular conduit conveying spermatozoa from the epididymis to the urethra, has been used as a model tissue for smooth muscle pharmacological and physiological advancements. Many drugs, notably α-adrenergic antagonists, have effects on contractility and thus normal ejaculation, incurring significant side effects for patients that may interfere with compliance. A more thorough understanding of the innervation and neurotransmitter pharmacology of the vas has indicated that this is a highly complex structure and a model for co-transmission at the synapse. Recent models have shown clinical scenarios that alter the vas contraction. This review covers structure, receptors, neurotransmitters, smooth muscle physiology, and clinical implications of the vas deferens.

## Introduction

In the treatment of male sexual disorders, focus has often been on erectile disorders and premature ejaculation (PE), the latter probably the most common disorder of male sexual function (Abdel-Hamid et al., 2009). Ejaculation consists of two distinct phases, emission and expulsion. Emission denotes the ejection into the posterior urethra of spermatozoa mixed with products secreted by accessory sexual glands. During the emission phase, both epithelial secretion and smooth muscle cell contraction take place throughout the seminal tract in a sequential manner. The function of the vas (ductus) deferens is to convey spermatozoa from the epididymis to the urethra. During emission, its coordinated muscular contractions propel the spermatozoa toward the urethra. However, the vas does not serve only as a conduit, but also contributes to secretion of fluid for sperm transport and possibly to resorption of spermatozoan remnants from the duct lumen. Adrenergic mechanisms play a major role for vas smooth muscle contraction, but many substances are capable of altering its contractility by modulating neurotransmitter release or the basal tone of the smooth muscle layers. Interference with the contractile function by, e.g., metabolic disorders and drugs used for lower urinary tract disorders, may lead to ejaculatory dysfunction, and ultimately anejaculation. The mechanisms regulating the contractile behavior of the vas may therefore be of interest as targets for drugs meant for control of ejaculation (e.g., contraception). In addition, these mechanisms may have general physiological/pharmacological interest since the isolated vas deferens has proven to be one of the most useful preparations for the study of basic physiological mechanisms and the effects of drugs. It has been used to study the electrophysiology of the smooth muscle myocytes and the release and inactivation of neurotransmitters, receptors and receptor-mediated mechanisms, and signaling pathways.

The present review gives an update on some of the mechanisms involved in the generation, propagation, and transduction of signals in the vas deferens. Some examples of the clinical consequences of interference with its contractile function are also given.

## General structure of vas deferens

**Figure 1:**
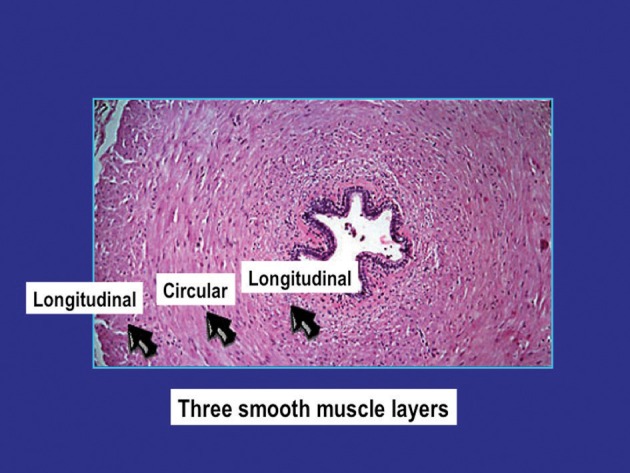
**Structure of the human vas deferens**.

The general structure and function of the vas deferens from humans and different animal species have many similarities (Steers, 1994; Dixon et al., 1998; Kaleczyc, 1998; Westfall and Westfall, 2001; Burnstock and Verkhratsky, 2010). The vas is a tubular structure consisting of a muscle coat, an inner mucosa and an outer adventitia. The smooth muscle coat, which may have a thickness of 1–1.5 mm, consists of a circular layer surrounded by inner and outer longitudinal layers. The circular layer is the most prominent and forms a tightly wound spiral, whereas the longitudinal layers are formed by muscle bundles slightly helical in their arrangement. The outer longitudinal smooth muscle cells are up to 30–40 μm in length and 2–5 μm in diameter (Figure 1). Each smooth muscle cell is closely associated with 6–12 other cells, with gaps as close as 15–20 nm (Elbadawi and Goodman, 1980). The cells are electrically coupled allowing electronic spread and depolarization to travel from one cell to the next. This intercellular coupling can be suppressed by heptanol (Manchanda and Venkateswarlu, 1997, 1999), believed to interact with gap junction function (Christ, 1995).

The lumen of the vas deferens is lined by columnar epithelial cells with microvilli extending into the lumen (Dixon et al., 1998). Blood supply comes from the inferior vesical artery. The vas is innervated by autonomic postganglionic nerve fibers originating primarily from neurons in pelvic ganglia, and to a lesser extent, from neurons in the caudal mesenteric ganglion and sympathetic chain ganglia, and also by sensory nerve fibers arising from dorsal root ganglia (Kaleczyc, 1998; Kihara et al., 1998; Burnstock and Verkhratsky, 2010). In rodents the hypogastric nerve provides bilateral innervation to the vas, and contractile responses can be elicited with hypogastric stimulation from either side (Kihara et al., 1996; Harji et al., 1998).

## Signal generation

### Autonomic effector mechanisms

#### Adrenergic nerves

Adrenergic nerves are the most common among the nerve fiber groups supplying the mammalian vas deferens. Early studies using fluorescence histochemistry and biochemical detection of catecholamines have revealed numerous adrenergic nerve fibers innervating the vas of many mammalian species including the rat, guinea-pig, rabbit, cat, opossum, bull, and pig. The vas deferens of man, other primates, dog, and possibly the fox receives a less dense adrenergic innervation than that of other species (see overviews by Dixon et al., 1998; Kaleczyc, 1998). More recent immunohistochemical investigations have confirmed the results of the histochemical studies. Kaleczyc et al. (1997), showed in the pig vas deferens, similar to what has been found in other mammals, that the adrenergic nerves were distributed in the lamina propria and throughout the circular and longitudinal muscle layers. In the lamina propria, the adrenergic axons formed a loose network with the nerve terminals sometimes found beneath, but never penetrating into, the epithelium. In the muscle layers, the nerves were usually more numerous and run chiefly along the smooth muscle cells.

There is morphological, physiological, and pharmacological evidence that the adrenergic neurons supplying the mammalian vas deferens can utilize adenosine triphosphate (ATP) and/or a related purine as a possible co-transmitter with noradrenaline (NA) (see below).

**Figure 2:**
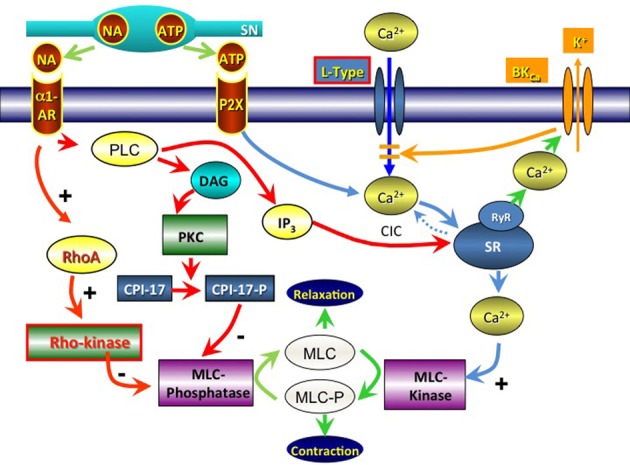
**Signal pathways involved in contractile activation of the vas deferens via noradrenaline (NA; α_1_-Adrenoceptors) and adenosine triphosphate (ATP; P2X receptors).** SN, sympathetic neuron; PLC, phospholipase C; DAG, diacylglycerol; PKC, protein kinase C; RhoA, ras homolog gene family, member A; CPI-17, protein phosphatase 1 regulatory subunit 14A; MLC, myosin light chain; IP3, inositol trisphosphate; SR, sarcoplasmic reticulum; CIC, calcium-induced calcium release; RyR, ryanodine receptor; BKCa, big K+ activated Ca^2+^ channel; L-type, L-type voltage dependent Ca^2+^-channel.

In human vas, varicose nerve terminals able to take up and bind quinacrine have been demonstrated. These nerves may represent purinergic nerves (Alm, 1982). It may be assumed that these nerves also contain NA, but this does not seem to have been established (Figure 2).

There is also evidence suggesting the coexistence of neuropeptides in noradrenergic nerve fibers supplying the mammalian vas deferens. Although there may be species differences, many noradrenergic nerves innervating the vas deferens muscle coat also express immunoreactivity to somatostatin, Leu-enkephalin, and neuropeptide Y (Kaleczyc, 1998; Burnstock and Verkhratsky, 2010). The majority of adrenergic (tyrosine hydroxylase-immunoreactive: IR) nerves supplying the muscle of the human vas deferens (Jen et al., 1997) were shown to contain neuropeptide Y.

#### Cholinergic nerves

Although the histochemical localization of acetylcholinesterase (AChE) is thought not to be specific for cholinergic nerve fibers (Lincoln and Burnstock, 1993), most information on cholinergic innervation of the vas is based on this methodology. The general impression is that cholinergic (AChE positive or choline acetyltransferase IR) nerve fibers supplying the mammalian vas deferens are fewer than the adrenergic ones and are mostly concentrated within the lamina propria (in contrast to adrenergic nerves that are present especially in the muscle coat). Such an innervation pattern has been found in many mammals including the rat, guinea-pig, dog, bull, monkey, and man (for review, see Kaleczyc, 1998; Burnstock and Verkhratsky, 2010).

The functional significance of cholinergic input to the innervation of the vas deferens has not been established. Sjöstrand (1962) suggested that the main action of the cholinergic innervation was to suppress adrenergic neurotransmission. In addition to such a function, there is some evidence that cholinergic nerves may act on the epithelial cells of the vas deferens (Sjöstrand, 1965), which may be responsible for fluid resorption from the lumen or for active secretion of certain components necessary for sperm maturation. Cholinergic nerve fibers supplying the mammalian vas deferens contain neuropeptides, particularly vasoactive intestinal polypeptide (VIP), NPY, and/or somatostatin, as well as other active substances such as nitric oxide synthase (Kaleczyc, 1998).

#### Afferent nerves

Neurons in the dorsal root ganglion contain a number of different substances including tachykinins, especially substance P (SP), and calcitonin gene-related peptide (CGRP). The mammalian vas deferens is supplied with some CGRP- and/or SP-IR nerve terminals that are presumed to derive from DRG (see, Kaleczyc, 1998), which is consistent with observations of Kolbeck and Steers (1993) in the rat that many DRG neurons project to the vas deferens. In the pig, double-labeling immunofluorescence has revealed almost a complete colocalization of SP and CGRP in some nerve fibers supplying the vas deferens (Kaleczyc et al., 1997). SP/CGRP-IR nerve terminals were located mainly in the longitudinal muscle layer where they sometimes appeared as very long, fine varicose fibers running parallel to the smooth muscle bundles. In the lamina propria, these fibers were occasionally discovered. CGRP-IR fibers supplying the vas deferens have been found in some other mammals including the guinea-pig, rat, and man. SP-IR nerves have been revealed in the mouse, guinea-pig, cat, rabbit, and man, but are absent from the vas deferens of the rat (see Kaleczyc, 1998; Burnstock and Verkhratsky, 2010). GCRP, widely distributed in peripheral and central sensory afferents throughout the body, are released in the vas deferens from various stimuli, notably capsaicin (Sheykhzade et al., 2011), which induces GCRP release through the transient receptor potential cation channel vanilloid subfamily member 1 (TRPV1) agonism. As a brief review, transient receptor channels are omnipresent in the body, typically allowing various cation passage with appropriate stimuli (Nilius et al., 2007). In the vas, vanilloid are the only subtypes to our knowledge.

#### Responses to nerve stimulation

Electrical field stimulation of the sympathetic nerves in the nonhuman vas deferens results in a contraction with two distinct components. The twitch or phasic component is transient, while the secondary tonic component is maintained for the duration of the stimulation. These biphasic responses have been found in the guinea pig, rat, mouse, and rabbit vas deferens (Ambache and Zar, 1971; Swedin, 1971, see review by Westfall and Westfall, 2001). Ambache and Zar (1971) suggested that the biphasic response was due to the involvement of a second neurotransmitter, and the inability of phentolamine and pretreatment with reserpine to block the phasic portion of the neurogenic response (Swedin, 1971) supported this suggestion. Burnstock (1972) proposed that the second transmitter was ATP, and Westfall et al. (1978) first demonstrated that stimulation of the vas deferens results in the release of purines. Since then, neuronal release of endogenous ATP and NA has been convincingly confirmed in several species using various techniques (see Westfall and Westfall, 2001). In the human vas, adrenergic mechanisms were considered primarily responsible for contraction of the smooth muscle, since the response to nerve stimulation was more or less completely blocked by α-adrenoceptor (AR) antagonists (Figure 2) (Anton and McGrath, 1977; Hedlund et al., 1985; Smith and Bray, 1990; Steers, 1994). However, Banks et al. (2006) demonstrated that the human vas deferens smooth muscle contracts in response to both adrenergic and purinergic agonists. They considered the adrenergic system functionally dominant, but that purinergic co-transmission was also functionally significant. While not certain, there have been studies suggesting the presence of pacemaker cells in the vas that initiate spontaneous contractions in a similar manner to the Interstitial Cells of Cajal (Metzger et al., 2008; Burnstock and Lavin, 2002). These cells may be *c-kit*^+^ interepithelial cells.

It has not been definitely established whether ATP and NA are co-stored and released from the same vesicles or stored and released from different vesicles. As pointed out by Knight et al. (2003), four possible scenarios for the storage and release of these two neurotransmitters exist: (1) ATP and NA may be stored and released from the same vesicles, (2) ATP and NA may be stored and released from separate vesicles, (3) ATP and NA might be stored and released from different sets of varicosities, (4) ATP and NA may be stored and released from the same vesicles but in different proportions in different varicosities. The concept of separate storage and differential release of ATP and NA seems to have growing support (Westfall et al., 2002). Additional studies indicate that the epithelium may play a role in the regulation of vas contractility. Ruan et al. (2008), demonstrated that exogenous ATP could inhibit EFS smooth muscle contraction in an epithelium dependent mechanism, likely through its induction of PGE_2_ synthesis. This was posed to be due to P2Y receptor activation by ATP, and calcium release from epithelium, which leads to cAMP-dependent K+ channel activation and membrane hyperpolarization of smooth muscle.

While ATP and NA are the primary affectors released from nerve terminals upon stimulation, other neurotransmitters have been posed to influence the neuromuscular relationship in the vas deferens. Li et al. (2003, 2006) and Hu (2007) have demonstrated that histamine coexists with NA in sympathetic nerves, is released with nervous stimulation, and may have sympathetic affects.

#### Postjunctional receptor mechanisms

As indicated above, contraction of the smooth muscle myocytes in the nonhuman vas deferens is elicited by at least two neurotransmitters, NA, which evokes a contraction mediated by α_1_-Adrenoceptors (Minneman et al., 1988; Honner and Docherty, 1999), and ATP, which evokes a faster contraction mediated by ligand-gated P2X_1_-receptors (Liang et al., 2000; Mulryan et al., 2000). Neurotransmitter release from sympathetic varicosities is highly intermittent (Brock and Cunnane, 1988), and non-uniform between varicosities (Lavidis and Bennett, 1992).

#### Purinergic receptors

Genetic as well as pharmacological and developmental studies provide strong evidence that P2X1 receptors, probably forming homomeric channels, are primarily responsible for fast purinergic transmission in the mouse vas deferens (Liang et al., 2000, 2001; Mulryan et al., 2000). It has been suggested that P2X receptors in the mouse vas deferens and other sympathetically innervated smooth muscles exist in clusters beneath sympathetic varicosities (Barden et al., 1999). However, only a small proportion of the P2X_1_-receptors located on a smooth muscle cell contribute to spontaneous EJCs, suggesting a diffuse distribution of P2X_1_-purinoceptors on the smooth muscle myocytes (Liang et al., 2001). Immunostaining results by several groups also support the notion that P2X_1_-purinoceptors in the mouse vas deferens are diffusely distributed over the entire surface of the smooth muscle cells (Vulchanova et al., 1996; Lee et al., 2000; Liang et al., 2001). Recent analysis of the P2X_1_ receptor in human vas deferens (Amobi et al., 2012) indicated P2X1-purinoceptor stimulation elicits excitatory effects that lead to longitudinal muscle contraction. There was a secondary activation of 4-aminopyridine-sensitive (KV), and iberiotoxin-sensitive (BKCa) K+ channels. The contraction mediated by P2X1-purinoceptor stimulation was subcontractile in circular muscle due to the ancillary activation of BKCa channels. These differences in activation between longitudinal and circular muscle were considered to have functional implication in terms of the purinergic contribution to overall contractile function of human vas deferens. Amobi et al. (2012) also considered the modulatory effects of KV and BKCa channels following P2X1-purinoceptor activation to be pivotal in providing the crucial physiological mechanism that ensures temporal co-ordination of longitudinal and circular muscle contractility. Interestingly, the BKCa channels have also been found to mediate vas smooth muscle relaxation when stimulated with sodium hydrosulfide (NaHS) (Li et al., 2012). Li et al. demonstrated that NaHS induced relaxation did not involve the nitric oxide pathway, nor transient receptor potential channels.

The most conclusive evidence to date that the P2X_1_-receptor mediates the postjunctional excitatory response to ATP in the vas deferens comes from mice lacking P2X_1_-receptors. The vas deferens from P2X_1_-receptor^−/−^ mice did not respond to exogenously applied ATP or α, β-meATP, and these tissues lacked spontaneous and evoked EJPs (Mulryan et al., 2000). A consequence of this gene deletion was a 90% reduction in the fertility of male animals, which resulted from a low sperm count in the ejaculated semen. Thus mutant females did not become pregnant when mated with mutant males, but normal rates of conception were observed when they mated with wild-type or heterozygous males. The sperm from the mutant male mice was, however, viable and able to fertilize ova *in vitro*. Mulryan et al. (2000) suggested that selective pharmacological blockade of P2X1 receptors should produce a similar effect, and might thus provide the means for developing a non-hormonal male contraceptive pill. To this effect, sildenafil, known to reduce vas contractility has been posed to inhibit contractions by way of the purinergic receptor system (Bilge et al., 2005). In addition, agents that potentiate the actions of ATP at P2X1 receptors may be useful in the treatment of male infertility.

#### α-Adrenoceptors

The myocytes from both human and non-human vas deferens express both α_1_- and α_2_-ARs (Hedlund et al., 1985; Salles and Badia, 1991; Ventura and Pennefather, 1994). It has been suggested that contractions of rat vas deferens smooth muscle cells to exogenous NA or adrenaline are mediated predominantly by α_1*A*_-ARs (Aboud et al., 1993; Honner and Docherty, 1999; Campos et al., 2003), or the postulated α_1*L*_-AR in addition to α_1*A*_-ARs (Ohmura et al., 1992). In a study of rat vas deferens, Honner and Docherty (1999) found that contractions to exogenous NA were mediated predominantly by α_1*A*_-adrenoceptors, and contractions to endogenous NA by α_1*D*_-ARs. Cleary et al. (2004) confirmed that the predominant α_1_-AR in rat vas deferens is the α_1*A*_-AR, both in terms of ligand binding and contractions to exogenous agonists. The α_1*D*_-AR was only detectable by ligand binding following chemical sympathectomy, but seemed to be involved in NA-evoked contractions.

The human vas deferens can be contracted by NA; this effect is mediated by α_1_-ARs, and the motility of the vas deferens can be effectively inhibited by α_1_-AR antagonists (Holmquist et al., 1990). In the human vas deferens, Furukawa et al. (1995) reported that the contractile response to l-phenylephrine is mediated by the α_1*A*_-AR subtype, a finding confirmed by several other investigators. Using RNase protection assay, *in situ* hybridization, and a functional study, Moriyama et al. (1997) confirmed that both the epididymal and pelvic portions of the human vas contained α_1*A*_-ARs mediating the contraction of phenylephrine. This was also found by Amobi et al. (1999a,b), who demonstrated that contractions evoked by NA in both longitudinal and circular smooth muscle from human vas deferens are mediated via activation of α_1*A*_-ARs. However the involvement of α_1*A*_-AR variants, such as the α_1*L*_-AR subtype may explain demonstrated differences in effects on longitudinal and circular muscle between some α_1*A*_-AR antagonists.

α_1_-AR antagonists are extensively used in the treatment of hypertension and lower urinary tract symptoms associated with benign prostatic hyperplasia. Among the side effects, ejaculatory dysfunction occurs more frequently with drugs that are relatively selective for α_1*A*_-ARs compared with other drugs of this class. Sanbe et al. (2007) explored physiological contribution of each α_1_-AR subtype using α_1_-AR subtype-selective knockout (KO) mice (α_1*A*_-, α_1*B*_-, and α_1*D*_-AR KO mice). They found that contractile tension of the vas deferens in response to NA was markedly decreased in α_1_-AR KO mice, and this contraction was completely abolished in α_1_-AR triple-KO mice. This attenuation of contractility was also observed in the electrically stimulated vas deferens. They concluded that α_1_-ARs, particularly α_1*A*_-ARs are required for normal contractility of the vas deferens and consequent sperm ejaculation as well as having a function in fertility. These findings seem to be valid also for humans, and the functional and clinical importance of the α_1*A*_-AR in the vas can be illustrated by the effects of silodosin, which has a high selectivity for the this receptor (Yamada et al., 2001).

Fifteen healthy male volunteers (urologists) took silodosin or a placebo twice daily for 3 days in a randomized, double-blind crossover design (Kobayashi et al., 2008). When on silodosin, all the subjects had a complete lack of ejaculation. Three days after completion of silodosin, the mean ejaculatory volume recovered to the baseline level. There was no sperm in urine after ejaculation under silodosin administration in any volunteer, and it was concluded that the mechanism of ejaculatory dysfunction caused by silodosin was a loss of seminal emission (anejaculation). Nagai et al. (2008) performed a real-time observation of ejaculation by healthy males sing color Doppler ultrasound in three healthy males. They concluded that the mechanism of ejaculatory dysfunction after silodosin was intricately related to retrograde ejaculation (retrograde inflow of seminal fluid), insufficient contraction of the seminal vesicles, and insufficient rhythmic contraction of the muscles of the pelvic floor. In a double-blind crossover study (Shimizu et al., 2010), 50 healthy volunteer men were randomly assigned to receive either a single dose of silodosin or placebo with 3 days of washout before crossover. Subjects masturbated 4 h after administering agents. Eleven men overall (22%) on silodosin administration had less than a 50% decrease from baseline in the amount of semen. It was concluded that silodosin may adversely affect the subjective orgasmic function by causing an abnormal ejaculation with decreased (or no) semen discharge and a decrease in the number of bulbocavernosus/pelvic floor muscle contractions. Anejaculation rather that retrograde ejaculation was produced. This has been confirmed in a number of clinical studies on patients with lower urinary tract symptoms associated with benign prostatic hyperplasia where the rate of abnormal ejaculation has been up to 28% (Kawabe et al., 2006).

In human vas deferens, Birowo et al. (2010) found that phophodiesterase inhibitors (PDEs), such as rolipram and RO-1724 (PDE4), milrinone (PDE3), and sildenafil (PDE5) effectively antagonized contraction induced by NA—this was accompanied by an up to 2–8-fold increases in tissue cAMP concentrations. Sildenafil produced a 12-fold increase in the cGMP concentration of the preparations. Whether or not this inhibitory action has any effects on ejaculation in men taking, e.g., PDE5 inhibitors remains to be established.

#### Other receptors

Substances other than ATP and NA can influence the contractility of the vas deferens, presumably via receptors located on the smooth muscle. Muscarinic receptor stimulation (carbachol) causes an M_2_-receptor mediated contraction of the vas deferens (Eltze, 1994). Vasopressin contracts the human vas via stimulation of V_1_ receptors (Andersson et al., 1988). β_2_-Adrenoceptors can influence sympathetic neuroeffector transmission both prejunctionally, where they facilitate equally well the release of sympathetic cotransmitters (see below) and postjunctionally, where they inhibit smooth muscle contractions evoked by ATP (Todorov et al., 2001). Other established receptors include serotonin (5HT) have been demonstrated in several studies. Kose et al. (2012) showed that a rat varicocele model showed decreased contractile response to 5HT. Given that there are several other substances that can modify the vas contractile response, including neuropeptide Y (Torres et al., 1992), endothelin (Telemaque and d'Orleans-Juste, 1991), vasopressin (Medina et al., 1998), and angiotensin II (Ellis and Burnstock, 1989; Maletìnská et al., 1998), it is possible that there are still unidentified receptors in vas deferens smooth muscle, or that the identified ones are promiscuous in their agonist recognition.

#### Prejunctional receptors

In the vas deferens of humans and various animal species, it has been amply demonstrated that a number of prejunctional receptors can modulate the release of NA and ATP. As in many other tissues, adrenergic nerves in the vas have prejunctional α_2_-ARs which, when stimulated, reduce the release of NA. In the vas deferens of various species, including the mouse, rat, and guinea pig, stimulation of the prejunctional α_2_-ARs not only reduces the release of NA, but that of ATP as well (Sneddon and Westfall, 1984; Driessen et al., 1993). ATP can also produce an inhibition of transmitter release in vas deferens (Von Kugelgen et al., 1989; Forsyth et al., 1991). In the prostatic portion of the rat vas deferens, endogenous ATP was found to exert a dual and opposite modulation of NA release: an inhibition through activation of P2Y receptors with a pharmacological profile similar to that of the P2Y_12_ and P2Y_13_ receptors and a facilitation through activation of P2X receptors with a pharmacological profile similar to that of P2X_1_ and P2X_3_, or PX_2_/P2X_3_ receptors (Queiroz et al., 2003). Some of the effects of ATP may be due to formation of adenosine. Adenosine reduced the amount of nerve-stimulated 3H-NA release, suggesting the involvement of a prejunctional P1 receptor of the A1 type (Hedqvist and Fredholm, 1976). Adenosine A2A receptors were found to facilitate NA release by a mechanism that involves a protein kinase C-mediated attenuation of effects mediated by presynaptic inhibitory receptors, i.e., α_2_-ARs, adenosine A1 and P2Y receptors (Queiroz et al., 2003). Queiroz et al. (2004) found that adenosine A(2B) receptors are involved in a facilitation of NA release in the prostatic portion of rat vas deferens.

Many other prejunctional receptors in the vas deferens from various species have been found to affect neurotransmitter release including opioid, cannabinoid, bradykinin receptors Trendelenburg et al. (2000), the β_2_-AR (Driessen et al., 1993, 1996; Todorov et al., 2001), the cholinergic nicotinic receptor (Todorov et al., 1991; Von Kugelgen and Starke, 1991), the NPY receptor Y2 (Bitran et al., 1991), GABA_*B*_ receptor (Strobel et al., 1989), histamine receptors (Zamfirova and Todorov, 1995; Poli et al., 1994), and receptors for endothelins and natriuretic peptides (Mutafova-Yambolieva and Radomirov, 1993; Mutafova-Yambolieva et al., 1993).

#### Regional variation in purinergic and adrenergic responses

It is well established that various regions of the vas deferens respond differently to nerve stimulation and exogenous agonists (Ventura, 1998). Segments from both ends of the vas deferens respond to ATP and NA however, segments from the prostatic end are more responsive to ATP and segments from the epididymal end are more responsive to NA (French and Scott, 1983; Schomig et al., 1990; Sneddon and Machaly, 1992). The density of adrenergic nerves and catecholamine content is higher in the prostatic than in the epididymal part of the vas. However, no differences in the distribution of P2X_1_ receptors (Knight et al., 2003) were demonstrated in the mouse vas, or in α_1_-Adrenoceptors in the human (Hedlund et al., 1985) or rat vas (Salles and Badia, 1991; Ventura and Pennefather, 1994). There is, however, evidence in a rat model that the density and mRNA level of α1-receptors, as well as maximal response to phenylephrine in the epididymal vas may decrease with age (Yono et al., 2008). In the mouse, the difference in response to ATP was attributed to insufficient nerve-terminal release of ATP in the epididymal part (Knight et al., 2003). Terradas et al. (2001) confirmed that the two portions of rat vas deferens differed in the postjunctional sensitivity to NA. Western blot analysis indicated a smaller concentration of Gq/11 protein in the prostatic half, and the authors suggested that the different sensitivity to NA could be due to the higher availability of this sort of G protein in the epididymal portion. The functional importance of this regional variation remains to be established.

## Signal propagation/spread

### Electrophysiology

Burnstock and Holman (1961, 1966) made the first recordings of EJPs produced by sympathetic nerves innervating the smooth muscle of the guinea-pig vas deferens (see, Sneddon, 2000). This led to the identification of ATP as the mediator of EJPs in this tissue. The EJPs are mediated solely by ATP acting on P2X receptors leading to action potentials and a rapid phasic contraction, whilst NA mediates a slower, tonic contraction which is not dependent on membrane depolarization.

In single smooth muscle cells from the human vas, Park et al. (2004) recorded and characterized two types of Ca^2+^ currents, the L and T-type. The importance of L type Ca^2+^ currents for vas contractility is well established (Ohya et al., 2001; Shishido et al., 2009), whereas the role and action of the T-type currents are not well defined. Park et al. (2004) also characterized two types of K+ channel currents, namely BK_Ca_ and delayed rectifier currents. Voltage-gated K+ currents (a fast-inactivating transient current and a delayed rectifier current) have also been demonstrated in rat vas deferens smooth muscle cells (Harhun et al., 2003). Their physiological importance has not been established.

### Intercellular communication

Paton et al. (1976), using electron microscope, was unable to demonstrate gap junctions in the vas deferens. However, there are reasons to believe that the smooth muscle cells of the vas are electrically coupled. Neurogenic contractions such as those evoked in the guinea pig vas deferens by stimulation of adrenergic nerves, only a small proportion of cells are directly influenced by transmitter released from the sympathetic motor innervation, because only about a fifth of the cells receive direct innervation by close-contact axonal varicosities (Merrillees, 1968; Bennett, 1973), and because varicosities do not release transmitter in response to every invasion by the axonal action potential because of the low probability of evoked transmitter release (Cunnane and Stjarne, 1984; Brock and Cunnane, 1988). Therefore, spread and co-ordination of excitation from the few directly activated cells to other cells probably requires the involvement of gap junctions. As mentioned previously, in the smooth muscle cells of the vas deferens, EJPs are produced following stimulations of adrenergic nerves. EJPs are thought to reflect not just depolarization of the cell being recorded from, but the summed activity of several cells in the neighborhood, by virtue of intercellular electrical coupling (Cunnane and Manchanda, 1990).

Manchanda and Venkateswarlu (1997) investigated the effects on EJPs of heptanol, a presumptive gap junction blocking agent (Christ, 1995), with a view to determining the influence of intercellular electrical coupling on smooth muscle junction potentials. Heptanol abolished rapidly and reversibly the EJP of the guinea pig vas deferens. Further investigation showed that heptanol inhibited both EJP-dependent and non EJP-dependent contractions of the vas, and that a postjunctional site of action of heptanol, probably intercellular uncoupling of smooth muscle cells, contributed to the inhibition of contraction (Venkateswarlu et al., 1999).

## Signal transduction

In the vas deferens, as in other types of smooth muscle, the most commonly used explanation for excitation–contraction coupling in smooth muscle cells is an increase in intracellular Ca^2+^ through either L-type Ca^2+^ channels or the release of Ca^2+^ from intracellular stores (Berridge, 1993, 2008). It has been shown that blockade of L-type calcium channels by nifedipine abolishes the purinergic component of contraction in mouse vas deferens (Cleary et al., 2003), suggesting that activation of the P2X1 receptors are dependent on Ca^2+^ influx. Brain et al. (2003), investigating the sources and sequestration of Ca^2+^ to neuroeffector Ca^2+^ transients in the mouse vas deferens, suggested that Ca^2+^ stores initially amplify and the sequester Ca^2+^ that enters through P2X receptors.

The contractile response of rat vas deferens myocytes to exogenous NA has been reported to be associated with the efflux of Ca^2+^ from the sarcoplasmic reticulum (SR) rather than the influx of Ca^2+^ via the plasmalemma. α-1-Adrenoceptors couple with phospholipase C (PLC) (Summers and McMartin, 1993; Burt et al., 1998) which produces inositol (1,4,5)-triphosphate (IP_3_) and diacylglycerol (DAG) using phospholipids from the plasma membrane (Berridge and Irvine, 1984). IP_3_ induces Ca^2+^ release from the SR, allowing the activation of myosin light chain kinase (MLCK) with ultimate phosphorylation of myosin with subsequent smooth muscle contraction (Somlyo and Somlyo, 1994). Khoyi et al. (1988, 1993) suggested that the NA response relies mainly on intracellular Ca^2+^ and that nifedipine-sensitive calcium entry may function as a trigger for calcium-induced calcium release (CICR) from the SR. However, in the rat vas deferens, there is apparently little contribution to NA-induced contraction from intracellular stores, as exposing intracellular Ca^2+^ stores to ryanodine, cyclopiazonic acid or thapsigargin had little effect on NA induced contraction (Amobi et al., 1999a,b). Amobi et al. (1999a,b) suggested that the NA-induced contraction may involve an increase in the sensitivity of the contractile apparatus to Ca^2+^, possibly through a Rho-kinase-mediated pathway (Büyükafar et al., 2003). In brief, the Rho is a GTPase and its downstream protein Rho-kinase is a downstream protein that mediates calcium sensitivity of smooth muscles via inhibition of myosin phosphatase, effectively maintaining smooth muscle contraction (Sward et al., 2000; Fukata et al., 2001); Rho kinase (ROCK-2) is expressed in mouse vas deferens, and inhibitors of Rho-kinase reduce contractions induced by NA (Amobi et al., 2006), phenylephrine, ATP, and KCl (Büyükafar et al., 2003).

There may be differences in the mechanisms for mobilizing intracellular Ca^2+^ in the prostatic and epididymal parts of the rat vas. Amobi and Smith (1999) suggested that, during stimulation of the epididymal part, the SR functions mainly to buffer calcium entering through nifedipine-sensitive voltage-gated calcium channels. In contrast, in the prostatic part, the SR serves mainly as a source of calcium and contributes more to contractions evoked by higher concentrations of the agonist.

It has been established that Ca^2+^ sparks are local and due to transient Ca^2+^ release events from the SR through ryanodine receptors (Jaggar et al., 2000). A spontaneous Ca^2+^ spark in the superficial area activates BK_Ca_ channels nearby and induces membrane hyperpolarization, which reduces Ca^2+^ channel activity. BK_Ca_ channels and ryanodine receptors may co-localize densely at the junctional areas of plasmalemma and SR fragments, where Ca^2+^ sparks occur to elicit spontaneous transient outward currents (STOCs). In single smooth muscle cells of guinea-pig vas deferens, Ca^2+^ entry through voltage-dependent Ca^2+^ channels in the early stages of an action potential may evoke calcium induced calcium release from discrete subplasmalemmal Ca^2+^ storage sites and generate local Ca^2+^ transients that spread over the cell to initiate a contraction (Imaizumi et al., 1998). In addition, the subplasmalemmal Ca^2+^ transients activate BK channels nearby, which results in the activation of Ca^2+^-dependent K+ current, a major outward current responsible for action potential repolarization and afterhyperpolarization. These two local Ca^2+^ release events, Ca^2+^ sparks at rest and Ca^2+^ transients upon depolarization, share physiological roles to activate BK channels and induce membrane hyperpolarization.

Ohi et al. (2001) studied local Ca^2+^ transient and distribution of BK_Ca_ channels and ryanodine receptors in the guinea pig vas deferens myocytes. They found that a limited number of discrete SR fragments in the subplasmalemmal area play key roles in the control of BK_Ca_ channel activity by generating Ca^2+^ sparks at rest to activate STOCs. These fragments also generate Ca^2+^ transients presumably triggered by sparks during an action potential to activate a large Ca^2+^-dependent K+ current and also induce a contraction. White and McGeown (2003) found in guinea pig vas myocytes that IP_3_ receptors Ca^2+^ regulate store content and modulate Ca^2+^ sparks, and that blockade of these receptors increases SR Ca^2+^ store content promoting Ca^2+^ sparks and STOC activity.

Medina et al. (2010) investigated the effects of K+ channel inhibitors on ring segments of the epididymal part of the human vas deferens. They found that charybdotoxin and tetraethylammonium (inhibiting non-selectively BK_Ca_ and IK_Ca_ channels), but not iberiotoxin (inhibiting selectively BK_Ca_ channels), apamin (inhibiting SK_Ca_ channels) and glibenclamide (inhibiting ATP sensitive K+channels) increased contraction induced by NA and electrical field stimulation. Theys suggested that the effects of charybdotoxin were mediated via L-type Ca^2+^ channels and an increase in Ca^2+^ influx.

### Models of altered contractility

While an array of studies have attempted to understand the physiologic nature of the vas with chemical and electrical stimulation, several models have been created to understand how different clinically applicable scenarios influence the vas. In an attempt to understand the influence of acute ischemia, models of torsion using rats have demonstrated a decrease in contractile response in the ipsilateral vas deferens (Karacay et al., 2011) Interestingly, spontaneous hypertensive rats have shown an INCREASE in contractile response to EFS and NA (Katsuragi et al., 1991), and Gur et al. (2010) showed increased contractile respone to purinergic stimulation in L-NG-Nitroarginine Methyl Ester (L-NAME) induced hypertensive rat vas deferens. In conjuction with this study, Gur et al. (2010) also demonstrated that rats co-treated with sildenafil and L-NAME reversed this EFS and α−β-methylATP hypercontractile property of the vas deferens. Additionally, a varicocele model has resulted in a decrease vas contractile response (Ozen et al., 2007). Taken together, alterations in vas are clinically applicable, and may have implications for fertility, ejaculation abnormalities, and possibly vasectomy associated pain (Granitsiotis and Kirk, 2004; Tandon and Sabanegh, 2008). Posed mechanisms for this supersensitivity are thought to be denervation related, and range from increased receptor density (Hata et al., 1981), partial resting membrane potential depolarization (Fleming et al., 1973; Fleming, 1975; Fleming and Westfall, 1975; Hershman et al., 1992, 1993, 1995) and changes in intracellular secondary messenger transduction and calcium sensitivity (Minneman et al., 1988; Abraham et al., 2003; Quintas et al., 2005; Amobi et al., 2006).

### Conflict of interest statement

The authors declare that the research was conducted in the absence of any commercial or financial relationships that could be construed as a potential conflict of interest.
